# Comprehensive Identification and Characterization of Human Secretome Based on Integrative Proteomic and Transcriptomic Data

**DOI:** 10.3389/fcell.2019.00299

**Published:** 2019-11-21

**Authors:** Geng Chen, Jiwei Chen, Huanlong Liu, Shuangguan Chen, Yang Zhang, Peng Li, Danielle Thierry-Mieg, Jean Thierry-Mieg, William Mattes, Baitang Ning, Tieliu Shi

**Affiliations:** ^1^The Center for Bioinformatics and Computational Biology, Shanghai Key Laboratory of Regulatory Biology, The Institute of Biomedical Sciences and School of Life Sciences, East China Normal University, Shanghai, China; ^2^National Center for Biotechnology Information, National Library of Medicine, National Institutes of Health, Bethesda, MD, United States; ^3^National Center for Toxicological Research, Food and Drug Administration, Jefferson City, AR, United States

**Keywords:** secreted proteins, proteome, transcriptome, RNA-seq, human secretome

## Abstract

Secreted proteins (SPs) play important roles in diverse important biological processes; however, a comprehensive and high-quality list of human SPs is still lacking. Here we identified 6,943 high-confidence human SPs (3,522 of them are novel) based on 330,427 human proteins derived from databases of UniProt, Ensembl, AceView, and RefSeq. Notably, 6,267 of 6,943 (90.3%) SPs have the supporting evidences from a large amount of mass spectrometry (MS) and RNA-seq data. We found that the SPs were broadly expressed in diverse tissues as well as human body fluid, and a significant portion of them exhibited tissue-specific expression. Moreover, 14 cancer-specific SPs that their expression levels were significantly associated with the patients’ survival of eight different tumors were identified, which could be potential prognostic biomarkers. Strikingly, 89.21% of 6,943 SPs (2,927 novel SPs) contain known protein domains. Those novel SPs we mainly enriched with the known domains regarding immunity, such as Immunoglobulin V-set and C1-set domain. Specifically, we constructed a user-friendly and freely accessible database, SPRomeDB (www.unimd.org/SPRomeDB), to catalog those SPs. Our comprehensive SP identification and characterization gain insights into human secretome and provide valuable resource for future researches.

## Introduction

The secretome of an organism represents the proteins released by all types of cells/tissues of this organism (Chua et al., 2012). Secretory Proteins (SPs) are crucial for maintaining cell-cell communication, proliferation, metabolism (Zhang et al., 2014), and immune functions (Bauer et al., 2006). Notably, many SPs have been identified as important biomarkers for diverse cancers, and some of them could be therapeutic targets (Schaaij-Visser et al., 2013). Therefore, human secretome provides a valuable resource for diagnosis, prognosis, and treatment of diverse diseases especially cancers (Brown et al., 2013).

The strategies for identifying SPs can be mainly grouped into two different categories: proteomic identification and genome-based computational prediction (Hathout, 2007). The improvement of high-throughput liquid chromatography-coupled tandem mass spectrometry (LC-MS/MS) has allowed the identification of over 1000 proteins in a single experiment (Schaaij-Visser et al., 2013; Ichibangase and Imai, 2014; Li et al., 2017; Zhang et al., 2018), which empowers proteomic approach to be the mainstay in SP identification. However, only a small fraction of the potential SPs has been experimentally validated (Brown et al., 2013; Schaaij-Visser et al., 2013), due to the complexity of SP isolation and identification. For example, proteomic analysis of serum or plasma has been restricted by the fact that highly abundant proteins (such as albumin) represent up to 80% of the total proteins (Georgiou et al., 2001), making the majority of proteins with low abundance difficult to be detected. By contrast, genome-based computational prediction of SP is based on the hypothesis that most of SPs have an N-terminal signal peptide sequence which helps proteins to transport the endoplasmic reticulum (ER) lumen (Rapoport, 2007). Genome-based prediction has been widely used to decipher the secretome in many species such as human, pufferfish, and pig (Klee et al., 2004). Although previous studies tried to explore the human SPs (Clark et al., 2003; Chen et al., 2005), a large number of SPs remains to be identified and validated. Moreover, the exploration of human secretome at both transcriptome and proteome levels is still lacking, and the functions of SPs are also largely unknown.

Here we systematically explored human SPs based on the comprehensive protein set derived from UniProt (The UniProt C, 2017), Ensembl (Aken et al., 2016), AceView (Thierry-Mieg and Thierry-Mieg, 2006), and RefSeq (Pruitt et al., 2014) databases. A total of 6,943 high-quality SPs were identified and 3,522 of them are novel. We further validated and characterized SPs using a large amount of MS data and RNA-seq data collected from public databases. Most of our identified SPs have supporting evidence at protein and/or transcript levels. We also found that a significant fraction of SPs were detected in plasma, urine, cerebrospinal fluid, saliva, and pancreatic juice. Furthermore, we investigated the functional domains of human SPs using both known and *de novo* domain prediction approaches, and assigned protein domains to SPs. Importantly, we constructed a user-friendly database named SPRomeDB to catalog the diverse information of identified SPs, which provides a valuable resource for studying human secretome.

## Materials and Methods

### Collection and Integration of Human Core Protein Sequences

Human protein sequences derived from four main public resources were integrated: (Chua et al., 2012) The AceView human transcriptome and putative protein sequence database (Version 2010) provided 179,606 non-redundant protein sequences; (Zhang et al., 2014) the UniProt database (Version 2016_04) provided 42,103 (Swiss-Prot), and 117,522 (TrEMBL) non-redundant human sequences in FASTA, divided into canonical and isoform sequences; (Bauer et al., 2006) the RefSeq database provided 74,180 non-redundant protein sequences with gene annotations downloaded from NCBI (Release 75); (Schaaij-Visser et al., 2013) the Ensembl database provided 83,992 non-redundant protein sequences. In total, 330,427 non-redundant proteins were obtained as the core human protein sequences for further analysis.

### Prediction of the Secretome From Core Protein Sequences

Secretory proteins were identified by our tunneled analysis pipeline. Firstly, the secretory protein sequence set was predicted by using SignalP (Version 4.1) (Nielsen, 2017) that incorporates a prediction of cleavage sites and a signal peptide/non-signal peptide prediction based on a combination of several artificial neural networks. 330,427 core human protein sequences were used as input for SignalP and D cutoff ≥0.8 as a score of high quality level to select candidates for SPs. Secondly, putative SPs by SignalP were scanned by MitoFates (Fukasawa et al., 2015) (Version 1.1), TargetP (Emanuelsson et al., 2000) (Version 1.1), and MitoCarta (Calvo et al., 2016) (Version 2.0), together. All mitochondrial proteins are omitted out from the set of predicted secreted proteins (SPs) by SignalP. Thirdly, NucPred (Brameier et al., 2007) (Version 1.1), and PredictNLS (Cokol et al., 2000) (Version 1.0.20) were used to remove nuclear proteins. Fourthly, mitochondrial and nuclear proteins defined by WoLF PSORT (Horton et al., 2007) (Version 0.2) as took the first place in the prediction ranks were eliminated. Finally, TMHMM (Version 2.0c) and PredGPI (Pierleoni et al., 2008) (Web server) were utilized to predict transmembrane proteins and GPI-anchored proteins. Proteins that have no transmembrane helices or have one transmembrane helix located in non-N-term signal region and no anchoring signals are considered asSPs. The remaining predicted SPs were defined as SPs or the SPRome (also named as the secretome).

### The Genes Encode Secreted Proteins

The coding genes of SPs were searched according to the annotation information from UniProt, RefSeq, Ensembl, and AceView databases. First, all SPs were tried to map Ensembl genes. SPs were not mapped to Ensembl genes were annotated to RefSeq genes next. Finally, SPs were not annotated to both Ensembl and RefSeq genes were searched AceView genes.

### Gold Standard Secreted Proteins and Gold Standard Non-secreted Proteins

Proteins from Swiss-Prot database satisfying the following seven conditions were defined as gold standard secreted proteins (GSSPs): (1) annotated by the gene ontology (GO) terms GO:0005578 (extracellular matrix) and GO:0005615 (extracellular space) as well as their child terms; (2) exiting evidence at protein level; (3) has signal peptide; (4) no transit peptide; (5) no intramembrane region; (6) no transmembrane region; and (7) match “secreted” in “Subcellular location” term. Conversely, 1,110 unique protein sequences were selected as GSSPs.

Gold standard non-secreted proteins (GSNPs) were gained through querying “Subcellular location” term from UniProt database using keywords “cytoplasm” and “nucleus.” Finally, 9,778 unique protein sequences were defined as GSNPs.

### Identification of Novel Secreted Proteins

We defined SPs existing in at least one of four resources, including SPDI (Clark et al., 2003), SPD (Chen et al., 2005), MetazSecKB (Meinken et al., 2015), and the study by Diehn et al. (2000), as known SPs. The remaining SPs we considered as novel SPs. Detailed criteria of SP selection for these four resources are as bellow:

We downloaded secreted protein sequences from SPD and extracted human secreted protein sequences through matching species information.

MetazSecKB SPs are divided into four categories: Curated secreted - (querying “Subcellular location” term from the Swiss-Prot using “secreted” and “extracellular”), Highly likely secreted - (predicted by at least 3 out of 4 predictors), Likely secreted - (predicted by 2 out of 4 predictors), and Weakly likely secreted - (predicted by 1 out of 4 predictors). From this database, we selected curated and highly likely secreted protein sequences.

SPDI and the study by Diehn et al. (2000) identified membrane-associated/secreted genes, we selected secreted and transmembrane genes, and then obtained protein sequences by querying the UniProt database. Proteins without a signal peptide (annotated in UniProt database) and proteins with more than two transmembrane helices or one transmembrane helix not located in non-N-term signal region (predicted by TMHMM) were discarded.

### MS Data Resources Used as Proteomics Evidences of SPs

Various sources of MS data were integrated and used for evaluating each predicted secretory protein’s existence at protein level. The NCBI MS raw data were obtained from NCBI Peptidome. EBI MS raw data were downloaded from EBI PRIDE. NIST in-house raw data were provided by NIST. ProteomicsDB MS raw data were gained from ProteomicsDB (project ID: PRDB000042)^1^. Human cell lines MS raw data were achieved from ProteomicsDB (see text footnote 1, project ID: PRDB000035).

To handle such massive and diverse MS/MS experimental data, we built up an automatic analysis platform by integrating TPP (Deutsch et al., 2010) and OPENMS (Bertsch et al., 2011). Also two prevalent proteomics libraries, ProteoWizard (Kessner et al., 2008) and the PRIDE-tool, suite were employed to develop our own tools to harmonize the pipeline. Three well-optimized open source database search engines including X!Tandem-native, X!Tandem-Kscore, and OMSSA were applied. Single experiment searching results were validated by PeptideProphet at peptide-spectrum matches (PSMs) level, then by ProteinProhet at protein identification level. Overall false discovery rate was estimated by equal-sized decoy protein database searching.

As the first step, mass spectrometer output files were converted to mzXML using the related tools: OPENMS: FileConverter, ProteoWizard:Msconvert. Secondly, these files were run under three search engines and the results were converted to the pepXML file format. Thirdly, PeptideProphet was used to validate the search engine results and to model correct vs. incorrect PSMs. Fourthly, the datasets were validated at the peptide-identification level with iProphet. Finally, protein-level validation and protein inference were performed with ProteinProphet. Every experiment was computed by those steps.

### Supporting Evidences at Protein and/or Transcript Levels

Protein-level evidences came from MS data, neXtProt knowledgebase, UniProt, and the Human Protein Atlas (HPA) databases. MS data were processed as above mentioned; In neXtProt and UniProt databases, protein sequences with protein existence “Evidence at protein level” were chosen; From HPA database, we gained gene names with “Evidence at protein level” in “HPA evidence” term and then obtained protein sequences from UniProt database.

Transcript-level evidences were derived from RNA-seq data of ProteomicsDB, neXtProt knowledgebase, UniProt, HPA, and AceView databases. According to the central dogma of molecular biology, proteins exist protein-level evidences will also have transcript-level evidences. So, we selected proteins with protein existence “Evidence at protein/transcript level” form neXtProt, UniProt, and HPA databases using the same method above-mentioned. AceView database provides experimental information regarding the range of biological occurrence for each transcript, such as tissues, cell types or diseases. We extracted proteins coded by AceView cDNA transcripts expressed in at least one tissue resource/condition. Moreover, we downloaded RNA-seq data of Human BodyMap from ArrayExpress (accession no. E-MTAB-513). Before processing RNA-seq data, we built a merged human reference genome annotation file by filling genes annotated in AceView or RefSeq but not in Ensembl into intergenic and intronic regions of Ensembl provided reference genome. Then the downloaded data generated from 16 human tissues (thyroid, testes, ovary, white blood cells, skeletal muscle, prostate, lymph node, lung, adipose, adrenal, brain, breast, colon, kidney, heart, and liver) were separately aligned to Ensembl reference genome using STAR (Dobin et al., 2013) (version 2.5.2a). Next, we estimated the expression levels of the transcript with the program “rsem-calculate-expression” in RSEM (Li and Dewey, 2011) software (version 1.2.31) using the merged genome annotation file and transcripts whose TPM value more than 0.1 were thought to be expressed.

### Identification of Expression-Enriched Transcripts

For detecting expression characteristic of secretome at transcriptional level, we analyzed RNA-seq data from human early embryos and expression data from GTEx. Embryos data were downloaded from ArrayExpress (Parkinson et al., 2007) (accession no. E-MTAB-3929) and processed using aforesaid method. TPM value 0.1 was also the off of expressed or not.

Based on the expression data of embryos and GTEx, we performed expression-enriched analysis. We applied ANOVA analysis followed by Tukey’s range test (Tukey’s honest significance differences) for each transcript to assess significance of differences (fold change ≥ 4 and FDR < 0.01) among different embryo stages and different normal tissues.

### Differential Expression Analysis in Diverse Human Cancers

Raw counts of all genes in 13 different cancers of TCGA (The Cancer Genome Atlas) (number of normal samples ≥ 20) were downloaded from Genomic Data Commons (Grossman et al., 2016) (GDC)^2^. Differential expression analysis was performed by R package DESeq2 (Love et al., 2014).

### Gene Ontology and Pathway Enrichment Analysis

All GO enrichment analysis and KEGG pathway analysis in this article were carried out using WebGestalt (Wang et al., 2013). Only those GO terms and pathways with an adjusted *P* < 0.05 were considered statistically significant.

### *De novo* Domain Boundary Prediction

For SPs without known domains, we used a neural network method, PPRODO, to predict domain boundaries, with the position-specific scoring matrix (PSSM) generated from PSI-BLAST. The prediction accuracy of the method is about 70% when we used 0.25 as the cutoff for boundary score.

## Results

### Identifying “High-Quality” Human SPs From Human Proteome

**FIGURE 1:**
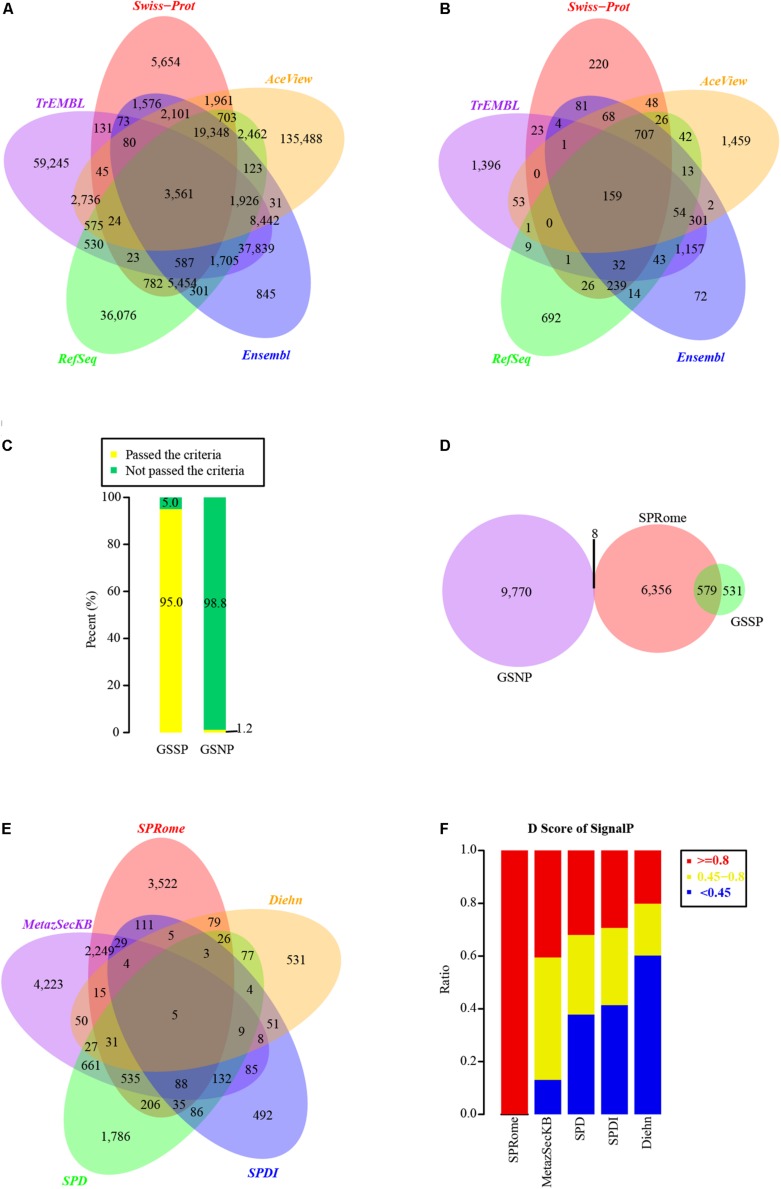
Identification and evaluation of SPs based on comprehensive human protein set. **(A)** Venn graph of human proteins used for SP identification in different databases. A total of 330,427 non-redundant proteins were integrated from Swiss-Prot, TrEMBL, RefSeq, Ensembl, and AceView databases. **(B)** The distribution of SPs identified in Swiss-Prot (1,635), TrEMBL (3,234), RefSeq (2,058), Ensembl (2,947), and AceView (2,934) databases. **(C)** The percentages of proteins passed and not passed the default SignalP cutoff (*D*-score >0.45) in GSSP and GSNP. **(D)** Comparison of our identified SPs with GSSP and GSNP. **(E)** Comparison of our identified SPs with other four known SP sets. **(F)** Distribution of SignalP *D*-scores in our identified SPs and other four SP sets.

By integrating the human proteins of UniProt (Swiss-Prot and TrEMBL), Ensembl, AceView, and RefSeq databases, a total of 330,427 non-redundant proteins were obtained for SP identification (Figure 1A). We first constructed a computational pipeline to identify the human SPs. The pipeline contained three main steps: (i) SP prediction (Supplementary Figure 1A); (ii) SP validation based on LC-MS/MS and RNA-Seq datasets from diverse tissues and cell lines; and (iii) functional annotation of SPs, including domain and family prediction as well as pathway enrichment analysis.

Considering that SPs generally possess a short peptide chain with a segment of hydrophobic sequences on the N-terminus of the nascent protein (Walter et al., 1984), we employed SignalP4.1 (Nielsen, 2017) to predict the potential SPs from 330,427 non-redundant proteins. In total, 31,332 SP candidates passed the default threshold *D*-score of 0.45, and 11,132 of them had *D*-score ≥0.8. We did not consider candidates with *D*-score <0.8 as SPs to increase the accuracy. Moreover, to minimize the false positives, 310 and 153 nuclear and mitochondrial proteins were excluded, respectively (see section “Materials and Methods”). Additionally, 3,231 proteins predicted as transmembrane proteins by TMHMM (Krogh et al., 2001) were also removed. Finally, 6,943 high-quality SPs were remained and 159 of them shared among Swiss-Prot, TrEMBL, Ensembl, AceView, and RefSeq (Figure 1B). Among those 6,943 SPs, 6,472 of them were encoded by 1,700 Ensembl, 1,244 RefSeq, and 177 AceView genes, while the rest of 471 SPs (13 SPs from Swiss-Prot and 458 SPs from TrEMBL) could not be mapped to known genes (see section “Materials and Methods”). Gene functional enrichment analysis showed that these SPs are mainly involved in the pathways of ECM-receptor interaction, Complement and coagulation cascades, Hematopoietic cell lineage, and Lysosome (Supplementary Figure 1B).

### Our Identified SPs Are With High Accuracy

To evaluate the accuracy and coverage of identified human SPs, we generated positive and negative protein datasets of human SPs. The GSSPs are the 1,110 known SPs derived from Swiss-Prot after a series of filtering (Supplementary Table 1). In contrast, 9,778 proteins defined as nuclear or cytosolic proteins, not secreted outside the cells, were used as the negative group (GSNPs, Supplementary Table 2). Strikingly, 95% of the GSSPs passed the default threshold of SignalP (*D*-score ≥0.45), whereas 98.8% of GSNPs were with *D*-score <0.45 (Figure 1C). To minimize false positives, we used a more stringent criteria of *D*-score >0.8, and removed nuclear, mitochondrial, and transmembrane proteins resulting in a list of high-quality SPs in aforementioned analyses. 6,943 high-quality SPs contain 579 GSSPs and only 8 of them (0.1%) were overlapped with the GSNPs (Figure 1D). Although we may miss a fraction of SPs, our criteria largely decreased the false positives. Some of the proteins annotated by Swiss-Prot as not secreted outside the cell may also be SPs, but current annotation methods are not able to effectively identify them. Accordingly, these SPs identified by us are with high-confidence.

We compared our SPs with published human SPs in other datasets, including SPDI (Clark et al., 2003), SPD (Chen et al., 2005), MetazSecKB (Meinken et al., 2015), and SPs identified by Diehn et al. (2000). Interestingly, half of our SPs were found in at least one of the four SP datasets (Figure 1E). Each of those SP sets contains a significant number of specific SPs and only 5 SPs were common among all SP sets (Figure 1E). Notably, only a small portion of proteins in those SP datasets had SignalP *D*-scores ≥0.8 (Figure 1F), indicating the lower quality of those SP datasets. Although MetazSecKB has relatively more human SPs than our SP set, the criteria used for SP identification in MetazSecKB is much looser. 3,522 SPs identified by us are novel, suggesting that the human SPs in previously existing databases are far from complete. These novel SPs are mainly enriched in the pathways of Complement and coagulation cascades, Hematopoietic cell lineage, Cell adhesion molecules (CAMs), and Lysosome (Supplementary Figure 1C). Accordingly, we largely extended the current human SP repository and increased the coverage of human SPs.

### Most of Our Identified SPs Have Protein and/or Transcriptional Evidences

**FIGURE 2:**
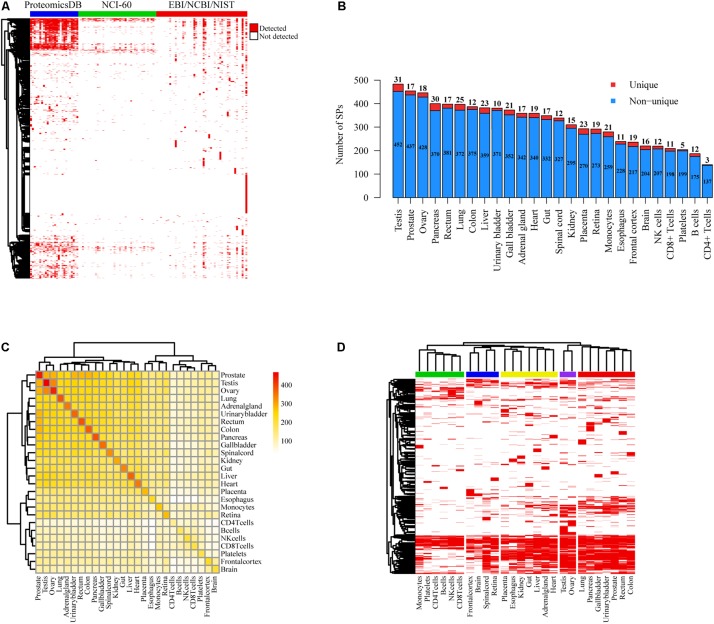
Protein level evidences of SPs supported by MS data. **(A)** Heatmap of SP presence in different tissues and/or cell lines based on the MS data of ProteomicsDB, NCI-60 cell lines, and EBI/NCBI/NIST database. Red represents the presence of SP, whereas white represents not. **(B)** Number distribution of detected SPs in distinct tissues and cells of ProteomicsDB project. The counts of unique and non-unique SPs were shown respectively. **(C)** Heatmap of shared SPs between each two different tissues or cells of ProteomicsDB project. **(D)** Clustering of different tissues and cells of ProteomicsDB project based on SP presence. Red stands for the presence of SP, whereas white represents not.

Since a large portion of SPs cataloged in public databases were derived from computational prediction without experimental validation, we used MS data collected from EBI (PRIDE) (Jones and Martens, 2010), NCBI (Ji et al., 2010) and NIST databases as well as the studies of ProteomicsDB (Wilhelm et al., 2014) and NCI-60 cell lines (Gholami et al., 2013) to confirm our SPs at the protein level (Figure 2A). Mass spectra from 28,251 experiments of over 40 different experimental conditions including diverse tissues and cell types were analyzed (Supplementary Table 3). We found that 2,461 (1,117 novel) and 1,616 (730 novel) SPs were separately matched with at least one and two unique mass spectra (FDR < 0.05) (Supplementary Figure 2A). The quantity of SPs detected in NCI-60 cancer cell lines were much less than those in tissues and cells of ProteomicsDB (Figure 2A). We also observed that 1,368 (1,107 of them have at least two supported unique peptides) and 1,591 (915 of them have ≥2 supported unique peptides) SPs could be detected with more than one unique peptide in the data sets of ProteomicsDB and EBI/NCBI/NIST, respectively (Supplementary Table 4). On average, 321 SPs were identified in each type of tissue or cells of ProteomicsDB and 75 SPs were detected in each condition of EBI/NCBI/NIST. Moreover, 932 of those 1,368 SPs were identified in at least two tissues of ProteomicsDB, while 757 of 1,591 were detected in more than one conditions of EBI/NCBI/NIST. Interestingly, testis, prostate, ovary, pancreas, and rectum are the top five tissues with the largest number of identified SPs, whereas the least number of SPs was detected in CD4T (Figure 2B). Specifically, we detected tissue-specific SPs based on MS data of ProteomicsDB, where the numbers ranged from 3 to 21 (Figure 2B). For example, we found that ACRBP, WNT5B, SERPINH1, MMP2, and CD4 are tissue-specific SPs for testis, prostate, ovary, pancreas, and CD4T. ACRBP is testis-specific SP, previous study has shown that ACRBP could be used to monitor the normal spermatogenesis of testes or *in vitro* development of germ cells (Kim et al., 2015). Analysis of circulating tumor cells (CTC) revealed that WNT5B is closely associated with prostate cancer (Chung et al., 2019). SERPINH1 has crucial function in collagen biosynthesis and is correlated with ovary development (Sato et al., 2002). It has been shown that MMP2 is associated with the progression of pancreatic cancer and could be a therapeutic target (Chen et al., 2019). CD4 is essential to initiate the early phase of T-cell activation. Moreover, prostate, testis, and ovary shared the largest number of SPs (Figure 2C). Notably, tissues can be grouped into different categories according to their SP presences, and neural tissues and genital tissues showed distinct SP expression patterns compared with other tissues (Figure 2D).

**FIGURE 3:**
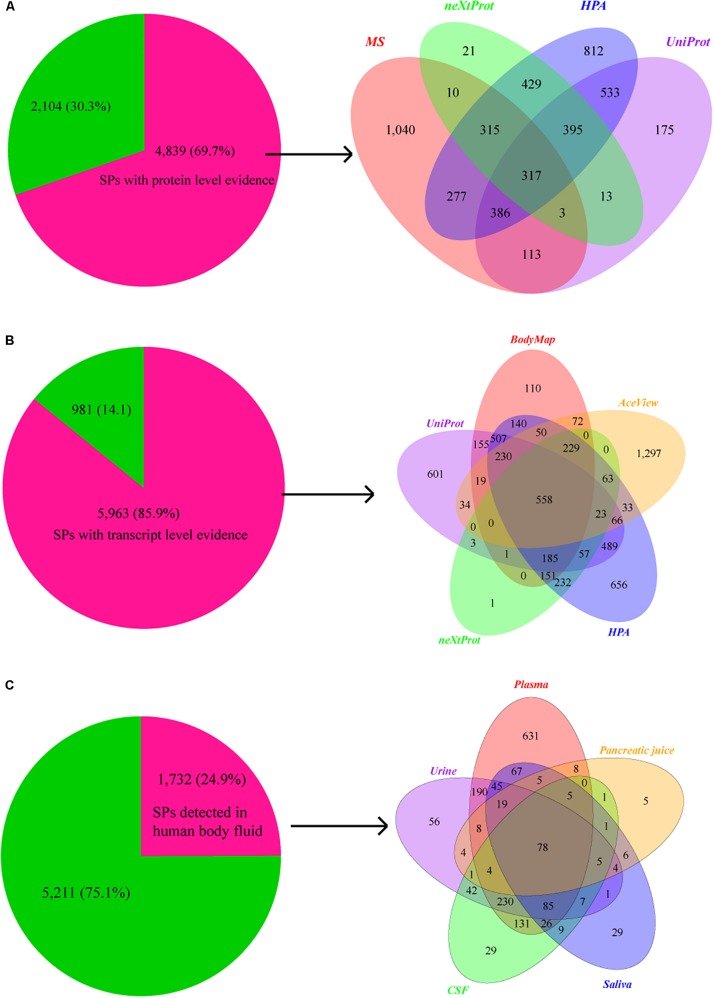
Supporting evidences of SPs at protein and transcript levels. **(A)** Pie chart shows the number and proportion of SPs that have supporting evidence at protein level, while Venn graph shows the distribution of protein level evidences for SPs in databases of neXtProt (2,461), UniProt (1,503), and HPA (1,935), as well as the MS data (3,464 SPs). **(B)** Pie chart shows the number and percentage of SPs that have supporting evidence at transcript level, while Venn graph shows the distribution of transcript level evidences for SPs in Human BodyMap project (2,407) and databases of UniProt (2,928), neXtProt (1,503), HPA (3,669), and AceView (2,674). **(C)** Pie chart shows the number and proportion of SPs that detected in human body fluids, while Venn graph shows the number of SPs detected in plasma (1,532), urine (779), cerebrospinal fluid (654), saliva (392), and pancreatic juice (154).

We then compared SPs with 37,089 proteins that have the protein level evidence curated from the publications in neXtProt knowledgebase (Gaudet et al., 2015). 1,503 of SPs (417 novel) have protein evidences. Furthermore, 1,935 SPs (532 novel) and 3,464 SPs (1,021 novel) are also annotated with protein evidence in the UniProt and the (Uhlen et al., 2015) HPA databases (Figure 3A). In total, 4,839 (1,902 novel) of 6,943 SPs have supporting evidences at the protein level. Considering the low detection rate of current MS technologies, we further checked the transcriptional evidence of SPs based on the RNA-seq data of human BodyMap, and the transcriptional evidences in neXtProt, UniProt, HPA, and AceView databases. Remarkably, 5,962 of those 6,943 SPs have the evidence at transcriptome level (Figure 3B). In total, 6,267 out of 6,943 (90.3%) of SPs have supporting evidence at protein and/or transcript levels, in which 1,902 and 2,659 novel SPs have the evidence at the protein and transcript levels, respectively. Therefore, the great majority of our identified SPs are supported by transcriptomics and/or proteomics data.

### A Large Portion of Our Identified SPs Are Detected in Body Fluids

Plasma is the body fluid commonly used in clinical diagnostics since it harbors proteins secreted from almost all tissues, and many plasma or serum proteins have been identified as potential biomarkers for diverse diseases including cardiovascular, autoimmune, infectious, and neurological disorders (Berhane et al., 2005; Agranoff et al., 2006). We found that 1,281 (332 novel) and 810 (72 novel) SPs are overlapped with the proteins in Plasma Proteome Database (Nanjappa et al., 2014) and Human Plasma Proteome Reference Set (Farrah et al., 2011) In addition, 121 (5 novel) SPs are in the urinary protein biomarker database (Shao, 2015). Moreover, 487, 434, 381, and 548 SPs were also detected the human urinary proteome described by other four publications (Adachi et al., 2006; Li et al., 2010; Marimuthu et al., 2011; Zheng et al., 2013), respectively. In cerebrospinal fluid, 309 and 624 SPs were detected by Kroksveen et al. (2011) and Schutzer et al. (2010), respectively. We also separately detected 302, 293, and 40 SPs using the data from three saliva related studies of Sivadasan et al. (2015), Sanguansermsri et al. (2018) and Zhao et al. (2018). Additionally, 149 and 34 SPs were identified based on the data of two pancreatic studies of Marchegiani et al. (2015) and Doyle et al. (2012).

In total, 1,532 (359 novel), 779 (226 novel), 654 (233 novel), 392 (71 novel), and 154 (31 novel) SPs identified by us were separately overlapped with the proteins detected in previous studies of plasma, urine, cerebrospinal fluid, saliva, and pancreatic juice (Figure 3C), resulting in a total of 1,732 SPs (486 novel). Therefore, the results indicate that our SPs can provide a valuable resource for clinical biomarker identification and diagnosis.

### The SPs Are Broadly Expressed in Early Embryos and Diverse Tissues at Transcriptional Level

**FIGURE 4:**
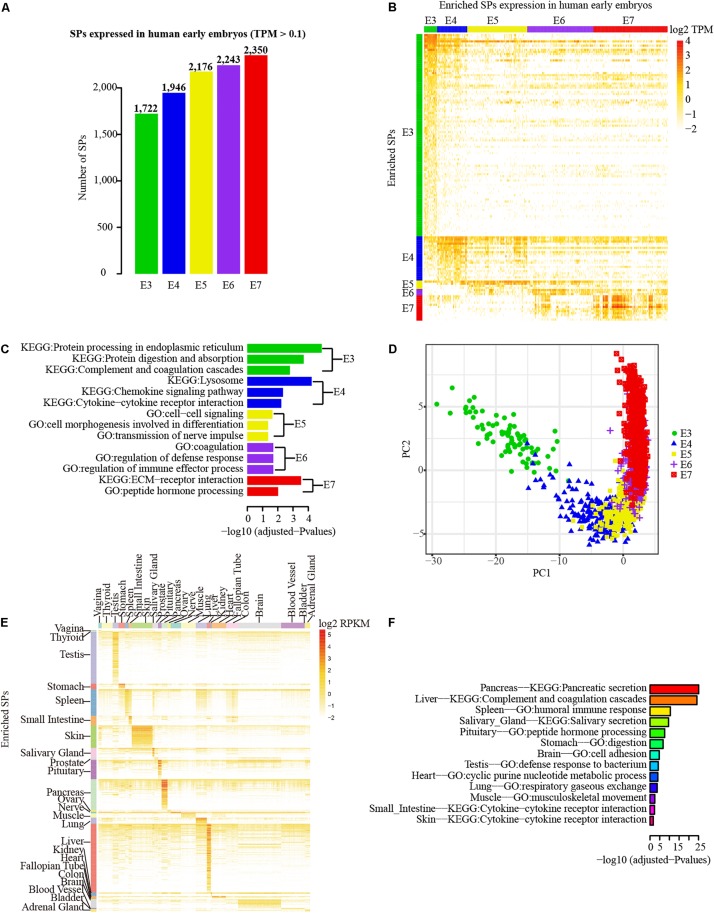
Transcriptional profiles of SPs in early embryos and different tissues. **(A)** Number distribution of detected SPs in each embryonic stage (0.1 TPM as cutoff). **(B)** Principal Component Analysis (PCA) of the samples different embryonic stages based on the SPs with enriched expression in each stage. **(C)** The SP genes that with enriched expression in each embryonic stage. **(D)** Functional enrichment analysis (GO and pathway) of expression enriched SP genes in different embryo stage. Green, blue, orange, purple and red separately represent stages from E3 to E7. **(E)** Expression enriched SP genes in different tissues of GETx project. **(F)** Functional enrichment analysis (GO and pathway) of expression enriched SP genes in different tissues.

To examine the transcriptional profiles of SPs, we investigated the expression patterns of SPs using the single-cell RNA-seq data of human early embryos. Because integrating the genes/transcripts annotated by different databases is challenging (Chen et al., 2013), we only combined genes from RefSeq and AceView databases that are located in the intergenic or intronic regions with Ensembl annotations. Excluding SPs that could not be accurately added into Ensembl annotation, 3,053 SPs (875 novel) were used for examining transcriptional expression profiling based on RNA-seq data. Using 0.1 TPM as cutoff, 2,753 (753 novel) of 3,053 SPs (90.17%) were detected in at least one stage of human early embryos at transcriptional level. Strikingly, the number of SPs detected in human early embryos was gradually increased from E3 (1,722 SPs) to E7 stages (2,350 SPs) (Figure 4A). However, expression enrichment analysis revealed that E3 stage has the largest number of SPs with enriched expression compared with other stages, whereas only three SPs (the least) showed enriched expression in E6 (Figure 4B, fold change ≥ 4 and adjusted *P* < 0.01). Intriguingly, functional enrichment analysis indicates that the enriched SPs identified in each stage were largely matched with the development features of early embryos (Figure 4C). The enriched GO terms and pathways are mainly related to the extracellular functional molecular, such as cytokines, chemokines, and extracellular matrix receptors, which are highly correlated with the development characteristics of human early embryos. For example, the enriched GO term of cytokine-cytokine receptor interaction is in line with the fact that embryonic cells of E4 stage start differentiation and cytokine is essential for cell differentiation. E7 embryonic cells prepare for implantation, thus the SPs with enriched expression in E7 stage were enriched in “ECM-receptor interaction” pathway. Notably, the cells of different embryonic stages could be clearly distinguished in PCA, and ordered by the developmental stages based on enriched SPs of each stage (Figure 4D). Consequently, the result suggests that SP genes are actively expressed in human early embryos.

To further explore the transcriptional expression profile of SPs in diverse tissues, 2,774 SPs (667 novel) that have Ensembl gene/transcript annotation were mapped to the expression table of Ensembl genes/transcripts obtained from GTEx project (Carithers and Moore, 2015). The SPs that could not be annotated to corresponding Ensembl genes/transcripts were not considered here. The great majority (2,625 out of 2,774) of SPs were expressed in at least one of 30 human tissues. Blood, brain, and adipose tissue were the top three tissues with the largest number of expressed SPs while fallopian tube was the least (Supplementary Figure 2B). Expression enrichment analysis showed that liver (138 SPs), testis (118 SPs), and pancreas (59 SPs) were the top three tissues with the largest number of enriched SPs, whereas no enriched SPs were found in whole blood, adipose, uterus, breast, esophagus, and cervix uteri based on the criteria of fold change ≥ 4 and adjusted *P* < 0.01 (Figure 4E). As expected, blood contains the largest number of detected SP, despite the lack of enriched SP expression, since whole blood may contain SPs secreted from diverse tissues. Furthermore, functional enrichment analysis of enriched SPs in different tissues revealed that the functions of SPs were closely associated with the functions of corresponding tissues (Figure 4F). For example, the SPs enriched in pancreas, spleen, and salivary tissues were mainly involved in pancreatic secretion pathway, humoral immune response function, and salivary secretion pathway.

### A Number of Our Identified SPs Are Functionally Important in Diverse Cancers

**FIGURE 5:**
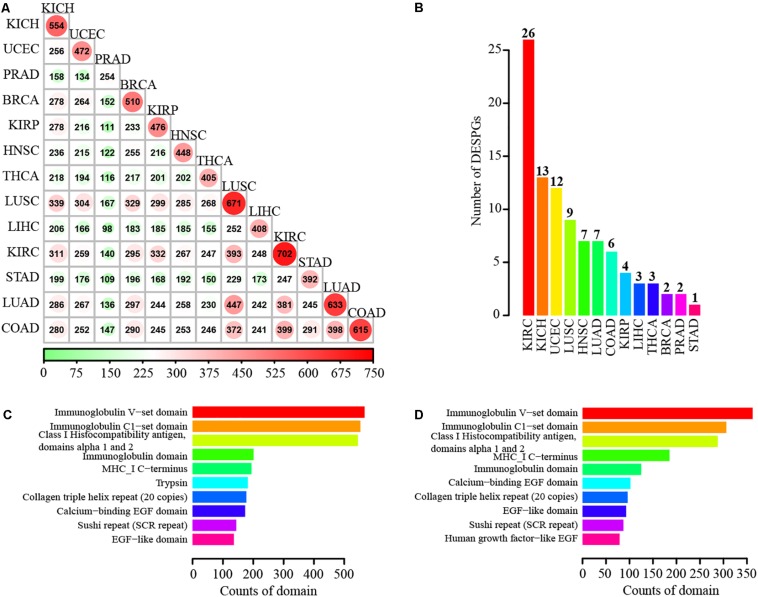
Differentially expressed SP genes in 13 different cancers and functional domain annotation of SPs. **(A)** Number of differentially expressed SPs shared by each two different cancers. **(B)** Number distribution of cancer-specific differentially expressed SPs. **(C)** Top 10 enriched domains of known SPs. **(D)** Top 10 enriched domains of novel SPs.

To investigate the expression changes of SPs at transcriptional level in cancers, we conducted differential expression determination between tumor and normal samples for 13 different cancers including breast invasive carcinoma, colon adenocarcinoma (COAD), lung adenocarcinoma (LUAD), prostate adenocarcinoma, and stomach adenocarcinoma of TCGA project (Evans and Relling, 1999). Hundreds of differentially expressed SP genes (DESPGs) were identified in each cancer type (Supplementary Figure 2C). Kidney renal clear cell carcinoma (KIRC) possessed the largest number of DESPGs, whereas prostate adenocarcinoma had the least (Figure 5A and Supplementary Figure 2C). Any two different types of cancers shared at least 98 DESPGs (Figure 5A). Moreover, 3 SPs were differentially expressed across 13 distinct cancers, while 90 DESPGs were found among > 10 cancers. Functional enrichment analysis of those 90 DESPGs showed that they were enriched in tumor-related pathways (such as Wnt signaling pathway, TGF-beta signaling pathway, focal adhesion, and ECM-receptor interaction) and secretory-related GO terms (e.g., extracellular structure organization and extracellular matrix organization) (Supplementary Figures 2D,E). Additionally, each cancer has its specific DESPGs (Figure 5B). Thus, DESPGs play important roles in cancers and each cancer has its specific pattern of DESPGs.

We further conducted Kaplan–Meier analysis to investigate whether the expression of those cancer-specific DESPGs was associated with the patients’ survival in corresponding tumors. Intriguingly, we detected 14 such cancer-specific DESPGs that their expression levels can be applied to significantly divide the patients into high-risk and low-risk groups (*P*-value < 0.05). The involved cancers and genes for these 14 DESPGs are Uterine Corpus Endometrial Carcinoma (UCEC) (e.g., GLB1, HSPA5, and PDIA3), KIRC (e.g., FUT11, GNRH1 and IFNGR2), Liver hepatocellular carcinoma (LIHC) (e.g., BGLAP and SSR2), Kidney renal papillary cell carcinoma (KIRP) (e.g., ADAM9 and TPST2), Thyroid carcinoma (THCA) (e.g., TGFBR1), LUAD (e.g., TAC4), Kidney Chromophobe (KICH) (e.g., BSG), and COAD (e.g., FUCA1) (see Supplementary Figure 3). Notably, FUT11 (Zodro et al., 2014), BGLAP (Yajima et al., 1989), SSR2 (Abdel-Hamid et al., 2014), TGFBR1 (Li et al., 2018; Tan et al., 2018), BSG (Tsai et al., 2007), and FUCA1 (Terraneo et al., 2013) have been reported to be associated with related cancer, but no studies showed the functions of other eight DESPGs in corresponding cancers. Therefore, these 14 cancer-specific DESPGs could be potential prognostic biomarkers for relevant tumors.

### The Great Majority of Our Identified SPs Possess Functional Domains

Since domains are the basic functional units of proteins (Deng et al., 2014), we identified domains in SPs. We scanned the SPs using InterPorScan (Jones et al., 2014) (version 57) based on eleven integrative protein family databases, including Pfam, CATH-Gene3D, PIRSF, PROSITE, HAMAP, PRINTS, ProDom, SMART, TIGRFAMs, SUPERFAMILY, and PANTHER. Strikingly, 89.21% of SPs and 90.15% of novel SPs were annotated with known domains. Moreover, most of SPs (82.33%) can be mapped to the domains in the Pfam database. Domains matched with SPs are mainly associated with immunity, such as Immunoglobulin V-set domain, Immunoglobulin C1-set domain, Class I Histocompatibility antigen, domains alpha 1 and 2, and Immunoglobulin domain (Figure 5C). The enriched families for 2,927 novel SPs include Immunoglobulin V-set domain, Immunoglobulin C1-set domain, Class I Histocompatibility antigen, domains alpha 1 and 2, MHC-I C-terminus, Immunoglobulin domain, Calcium-binding EGF domain, and so on (Figure 5D).

To characterize the protein domains in 749 (347 novel)SPs that have no assigned known domains, we conducted *de novo* domain boundary prediction by employing PPRODO (Sim et al., 2005). The majority of 749 SPs were assigned with 902 domain regions, with a cutoff of 0.25 for the boundary score (generally equates to a prediction accuracy of ∼70% and ∼75% for one-domain and two-domain chains). Specifically, 309 of 347 novel SPs were annotated with novel domains. Taking together, 6,845 of all the 6,943 SPs and 3,484 of the 3,522 novel SPs were annotated with known or novel domains, suggesting that those SPs identified by us are with functions.

### Our Identified SPs Are Freely Accessible in SPRomeDB Database

To provide a research resource for our identified SPs, we developed a user-friendly and freely available open access database namely SPRomeDB^3^. All data of human SPs are presented in SPRomeDB without restrictions. Users are able to conveniently browse and use the sources of SPs in the SPRomeDB. We believe that SPRomeDB is a valuable SP database to help researchers to gain insights into human SPs and conduct various related studies.

## Discussion

In this study, we systematically explored human SPs based on the non-redundant proteins integrated from UniProt, Ensembl, AceView, and RefSeq databases. Since the characteristics of SPs are complicated, we employed stringent cutoff to minimize the false positives. Although our stringent criteria missed a number of GSSPs, our identified SPs were with high-confidence and only 8 of them (0.1%) were overlapped with GSNPs. After a series of analyses, a total of 6,943 high-quality SPs were identified and 3,522 of them are novel, suggesting that the known human SP set was far from complete. Strikingly, most (89.21%, 6,194 out of 6,943) of our identified SPs were annotated with known protein domains, indicating that they could play important biological roles. By processing a large amount of MS/MS and RNA-seq data, we found that the great majority (90.3%) of SPs were expressed at protein and/or transcript levels, which further provides supporting evidences for those SPs. In order to facilitate SP researches, we constructed SPRomeDB database to enable users to freely accessible the resource of our identified SPs.

We observed that those SPs were broadly expressed in diverse tissues and cell types. Interestingly, the largest numbers of SPs were identified in testis, prostate, ovary, pancreas, and rectum, which is reasonable since that these tissues usually generate a lot of SPs to maintain their functions. Testis secretes hormones (primarily testosterone) and is the primary male reproductive organ, while ovary also produces the female hormones estrogen and progesterone are for female reproductive system. The main function of prostate is to secrete prostate fluid, one of the main components of semen. Pancreas produces insulin and other crucial enzymes as well as hormones for maintaining metabolic homeostasis. Rectum is the terminal segment of the digestive system, which also could secrete related proteins. In contrast, the smallest number of SPs was detected in CD4T cells. Although CD4T cells could secrete cytokines and chemokines to activate and/or recruit target cells, CD4T cells are not solid tissue and many SPs may not be captured by experiment.

Moreover, we observed that a significant fraction of SPs were with enriched expression in different stages of early embryos. Intriguingly, gene functional enrichment analysis indicated that some SPs were enriched in the pathways of ER processing in E3 stage and lysosome in E4 stage. We further compared our identified SPs, GSSPs, and GSNPs with the ER-resident and lysosomal proteins. The result showed that 15, 3, and 2 proteins were overlapped with the 56 ER-resident proteins from the study of Pehar et al. (2012) in our identified SPs, GSSPs, and GSNPs, respectively. We also obtained 452 lysosomal proteins from hLGDB database (Brozzi et al., 2013) and separately detected 61, 39, and 58 proteins in our detected SPs, GSSPs, and GSNPs. Therefore, some ER-resident and lysosomal proteins are SPs and the SPs with enriched expression in corresponding embryonic stages might be closely related to the development of different segments of the secretory pathway.

Additionally, we identified 14 cancer-specific DESPGs that their expression levels were significantly associated with the prognosis of eight tumors of UCEC, KIRC, LIHC, KIRP, THCA, LUAD, KICH, and COAD. Only six of them have been studied in relevant cancers. For example, a previous study indicated that FUT11 could be a potential biomarker for the progression of KIRC through meta-analysis (Zodro et al., 2014). SSR2 has been shown to be a reliable cancer biomarker for LIHC (Abdel-Hamid et al., 2014). TGFBR1 is a receptor of TGF-β ligands and could be correlated with thyroid tumorigenesis (Tan et al., 2018). However, the remaining eight cancer-specific DESPGs of GLB1, HSPA5, PDIA3, GNRH1, IFNGR2 ADAM9, TPST2, and TAC4) were not reported in any researches, which could be potential novel prognostic biomarkers in corresponding tumors.

Collectively, we systematically analyzed and characterized human SPs and identified 3,522 novel SPs, which largely extended the human SP repository. Most of our identified SPs contain functional domains and a number of them are closely associated with early embryonic development or the prognosis of different human cancers. Moreover, the user-friendly database SPRomeDB can provide valuable SP resource for future researches and clinic applications.

## Data Availability Statement

Publicly available datasets were analyzed in this study. This data can be found here: https://www.proteomicsdb.org/#projects/42, https://www.proteomicsdb.org/#projects/35, https://www.ebi.ac.uk/arrayexpress/experiments/E-MTAB-513/, https://www.ebi.ac.uk/arrayexpress/experiments/E-MTAB-3929/, and https://gtexportal.org/home/.

## Author Contributions

TS, GC, and PL designed the project. JC performed most of the analysis. GC and JC wrote the manuscript. PL, SC, and YZ performed the MS data analysis. HL implemented the database. DT-M, JT-M, WM, BN, and TS revised the manuscript.

## Conflict of Interest

The authors declare that the research was conducted in the absence of any commercial or financial relationships that could be construed as a potential conflict of interest.

## Footnotes

1 https://www.proteomicsdb.org

2 https://gdc.cancer.gov/

3 www.unimd.org/SPRomeDB
