# Supplementary material for: Comprehensive Identification and Characterization of Human Secretome Based on Integrative Proteomic and Transcriptomic Data
Source: Front Cell Dev Biol. 2019 Nov 21;7:299. doi: 10.3389/fcell.2019.00299 (PMC6881247; doi:10.3389/fcell.2019.00299)
Supplement: Supplementary file 5 [file Data_Sheet_1.DOCX]

Supplementary Material

## Supplementary Figures


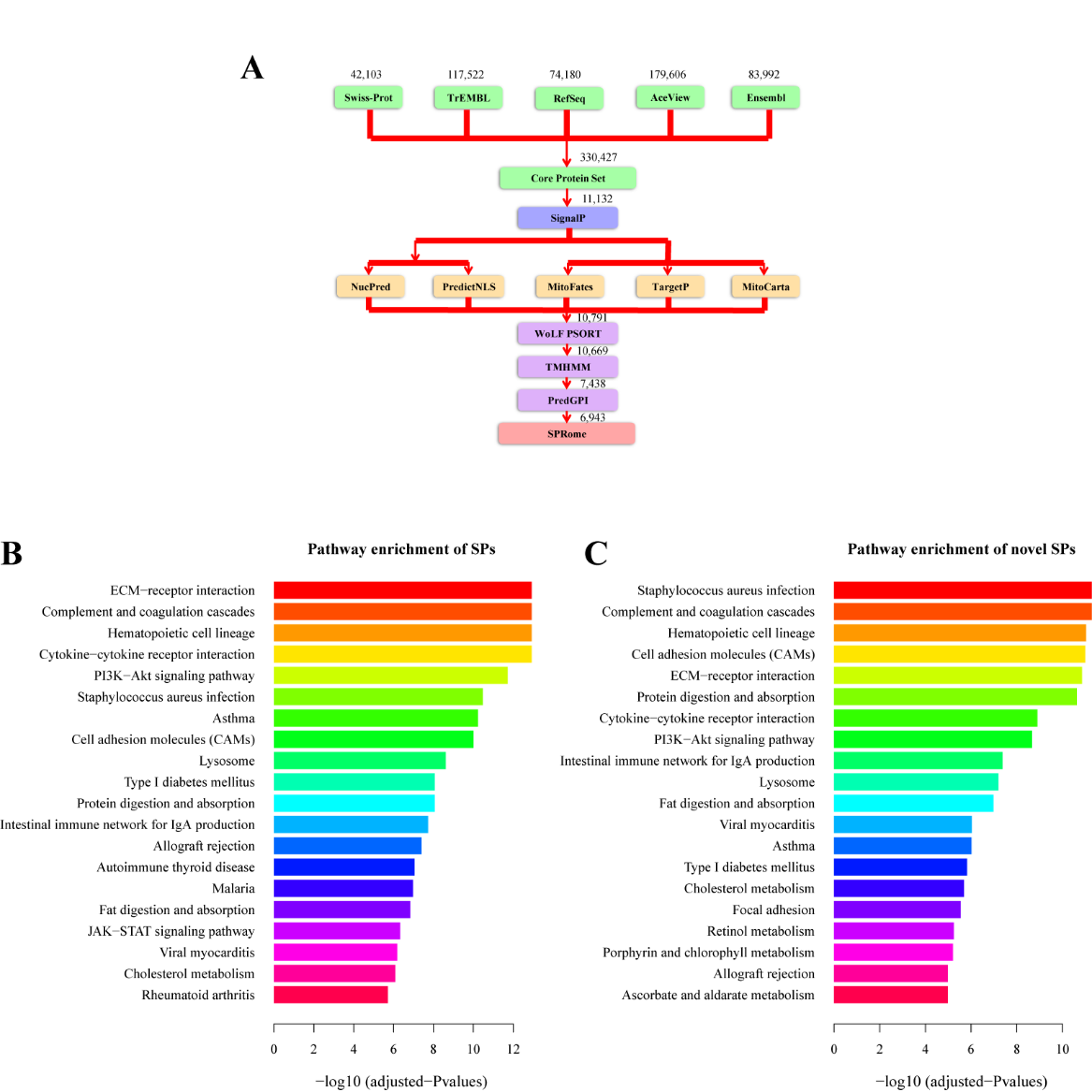


**Supplementary Figure 1.** (A) We built a computational prediction pipeline to predict the human secretome using various softwares and methods, combined with resourceful protein sequence datasets. Define core human proteome from main databases (Swiss-Prot, TrEMBL, RefSeq, Ensembl and AceView) and use SignalP to predict secretory proteins; after filtering out mitochondrial proteins (defined by MitoFates, TargetP, WoLF PSORT software prediction and MitoCarta database mining), nuclear proteins (defined by NucPred, PredictNLS and WoLF PSORT software prediction) , transmembrane proteins (predicted by TMHMM) and GPI-anchored proteins (predicted by PredGPI), we obtain high quality predicted secretome. (B) KEGG pathway enrichment analysis of SPs (C) KEGG pathway enrichment analysis of novel SPs.


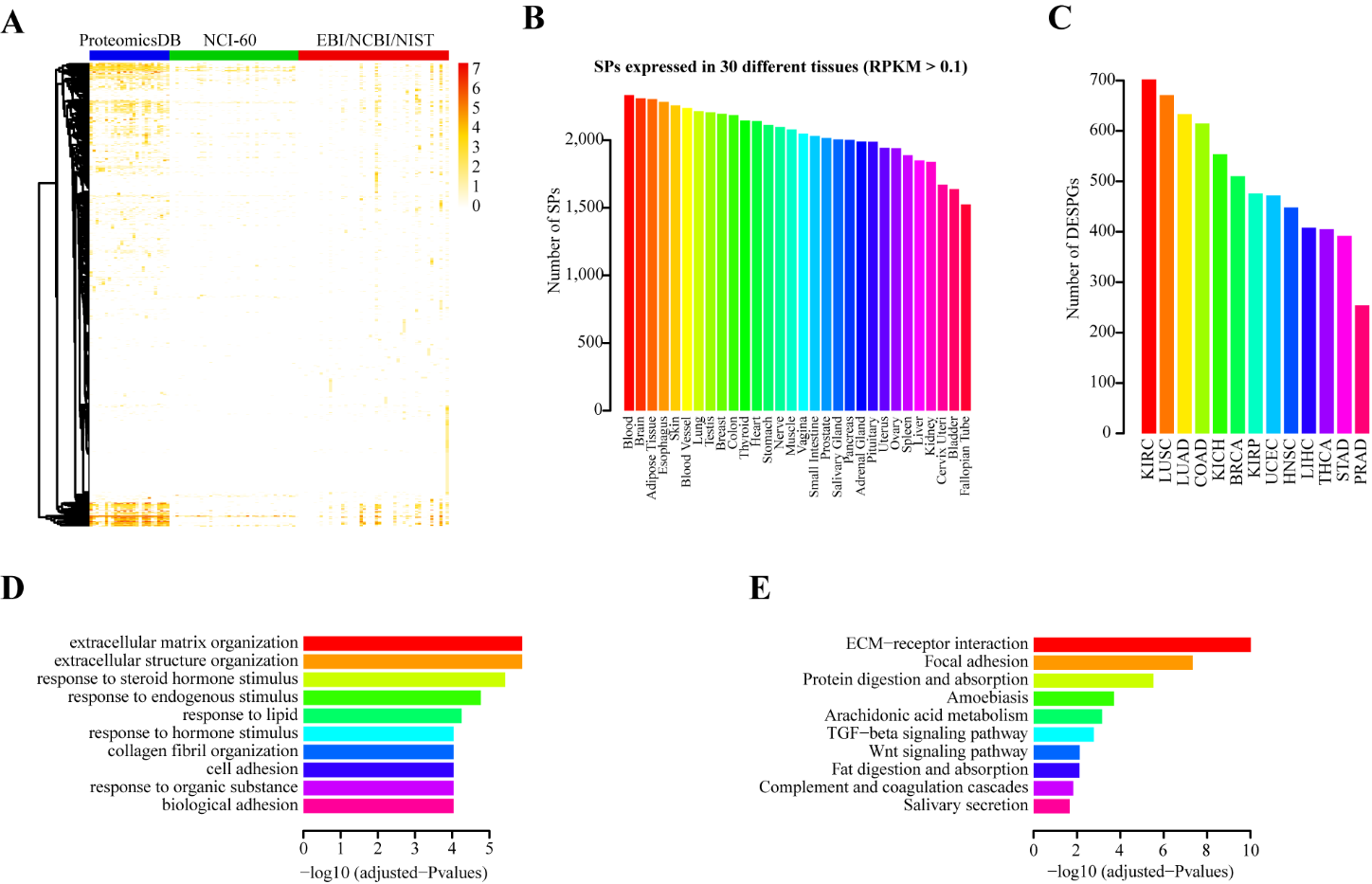


**Supplementary Figure 2.** (A) The number of detected unique peptides of SPs in MS data. The value was transformed by log2(number+1). (B) The number of expressed SPs in every tissue from GTEx project. Using RPKM 0.1 as cutoff for determining SPs express or not. (C) The number of DESPGs in different cancers. (D) GO enrichment analysis of 90 common DESPGs. (E) KEGG pathway enrichment analysis of 90 common DESPGs.


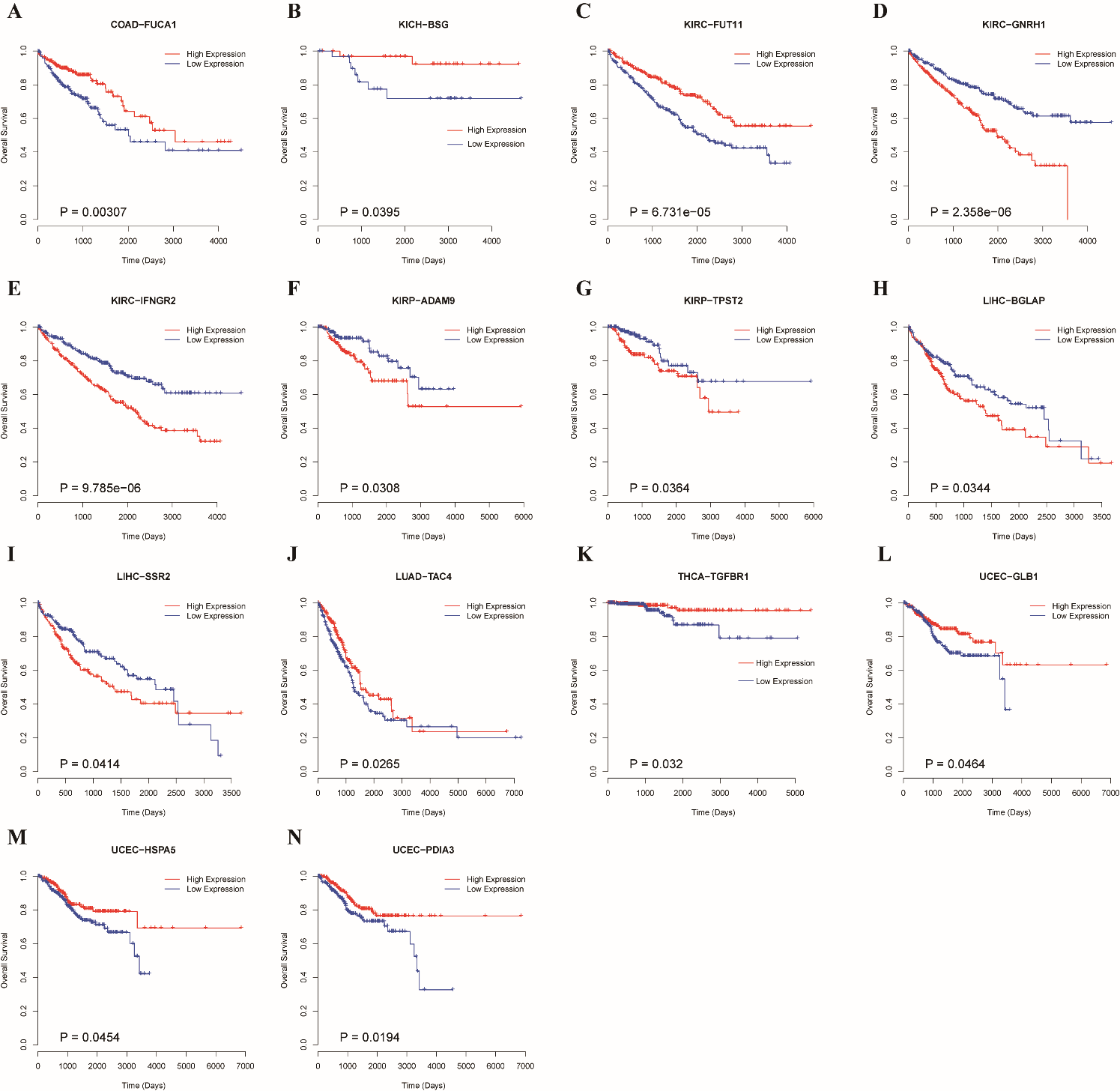


**Supplementary Figure 3. Prognostic analysis of cancer-specific DESPGs using the TCGA data.**
